# Simple sequence repeats and their expansions: role in plant development, environmental response and adaptation

**DOI:** 10.1111/nph.70173

**Published:** 2025-05-05

**Authors:** Sridevi Sureshkumar, Aaryan Chhabra, Ya‐Long Guo, Sureshkumar Balasubramanian

**Affiliations:** ^1^ School of Biological Sciences Monash University, Clayton Campus Melbourne VIC 3800 Australia; ^2^ State Key Laboratory of Plant Diversity and Speciality Crops/State Key Laboratory of Systematic and Evolutionary Botany, Institute of Botany, Chinese Academy of Sciences Beijing 100093 China; ^3^ College of Life Sciences, University of Chinese Academy of Sciences Beijing 100049 China

**Keywords:** epigenetic gene silencing, microsatellites, polyQ/polyglutamine, protein–protein interactions, repeat variability

## Abstract

Repetitive DNA is a feature of all organisms, ranging from archaea and plants to humans. DNA repeats can be seen both in coding and in noncoding regions of the genome. Due to the recurring nature of the sequences, simple DNA repeats tend to be more prone to errors during replication and repair, resulting in variability in their unit length. This feature of simple sequence repeats led to their use as molecular markers for mapping traits in diverse organisms. Advances in genomics, and the ever‐reducing costs of genome sequencing have empowered us to assess the functional impacts of DNA repeats. The variability in repeat lengths can cause phenotypic differences depending on where they are present in the genome. Variability in the repeat length in coding regions of genes results in poly amino acid stretches that appear to interfere with protein function, including the perturbation of protein–protein interactions with diverse phenotypic impacts. These are often common allelic variations that can significantly impact evolutionary dynamics. In extreme situations, repeats can undergo massive expansions and appear as outliers. Repeat expansions underlie several genetic defects in plants to diseases in humans. This review systematically analyses tandem DNA repeats in plants, their role in development and environmental response and adaptation in plants. We identify and synthesise emerging themes, differentiate repeat length variability and repeat expansions, and argue that many repeat‐associated phenotypes in plants are yet to be discovered. We emphasise the underexplored nature and immense potential of this area of research, particularly in plants, and suggest ways in which this can be achieved and how it might contribute to evolution and agricultural productivity.

## 摘要

重复序列在古菌、植物乃至人类等几乎所有生物基因组的一个普遍特征。无论是在编码区还是非编码区，重复序列都广泛存在。由于其内在的周期性排列特性，简单串联重复序列在DNA复制与修复过程中更易产生错误，导致简单串联重复序列位点的长度呈现高度多态性。这种遗传变异特性使其成为定位各种性状变异决定基因的分子标记。随着基因组测序技术的飞速发展与测序成本的持续降低，科学家得以深入探究简单串联重复序列的对生物学功能的影响。简单串联重复序列变异的对表型的影响依赖于其在基因组中的位置。当这种变异发生在编码区域时，会形成多聚氨基酸链(如多聚谷氨酰胺)，这类异常结构通过干扰蛋白质功能(如破坏蛋白质相互作用网络)产生多样化的表型效应。简单串联重复序列变异是常见的遗传变异，在生物进化中扮演着重要角色。在极端情况下，重复序列会发生远超群体正常长度范围的异常扩增。在植物中，这类扩增是多种遗传缺陷的根源；在人类中，则与疾病密切相关。本综述系统解析了植物简单串联重复序列的特征，探讨了其在植物生长发育、环境响应中的调控作用，及其作为适应性进化驱动因子的潜在机制。通过整合前沿研究成果，我们厘清了序列多态性与异常扩增的效应差异，指出植物中尚存大量未被揭示的简单串联重复序列相关表型。我们总结了该研究领域(尤其在植物中)仍存在广阔的探索空间，提出了可行的研究路径，强调了其突破性发现将为进化机制解析与农业生产力提升提供新思路。

**Table:** 

	Content	
	Summary	504
I.	Introduction	505
II.	Common allelic variation in repeat lengths vs rare repeat expansions	506
III.	Repeats in plant genomes	506
IV.	Variation in repeats contributes to phenotypic diversification	507
V.	Variation in polyQ stretches modulate the function of ELF3 protein	507
VI.	Variation at polyQ influences biochemical properties of the ELF3 protein	507
VII.	PFT1 – an example of evolutionary constraints on polyQ repeat number	508
VIII.	Variation in homopolymeric stretches of amino acids can affect protein–protein interactions	508
IX.	Variation in polyQ can affect subcellular localisation of the protein	509
X.	Allelic variation in polyQ contributed to evolutionary radiation	509
XI.	Repeats in noncoding regions	509
XII.	Repeat expansions	510
XIII.	A case of repeat expansion in plants	511
XIV.	Repeat expansions can give rise to small RNAs, which lead to epigenetic silencing	512
XV.	Evolution of repeats and repeat expansions	512
XVI.	DNA repeat variability and repeat expansions – an underexplored resource of genetic variation	513
	Acknowledgements	513
	References	514

## I. Introduction

Genomes of organisms are made up of specific sequences of four possible nucleotides. Given that there are only four possible nucleotides, often the sequences are repetitive in nature. During DNA synthesis by replication, DNA polymerases correct copying errors by recognising mismatches. However, repetitive sequence elements present a unique challenge, where the ability to detect the error is severely compromised. This leads to more variability in repetitive sequences in the genomes of organisms. Original observations on repetitive DNA were based on the reannealing kinetics of denatured DNA, which can be observed through the concentration of DNA that is reannealed as a function of time (CoT curves). Repetitive DNA will renature faster than unique sequences. In these analyses, in the pre‐sequencing era, repetitive DNA elements were recognised as DNA that is somewhat separated from the main DNA, both in their absorption spectrum and in their sedimentation gradient, and appeared as a minor ‘satellite’ band (Kit, 1961). The features of this satellite DNA were unclear at that time. Later, they were found to be repetitive sequences, which are faster in their re‐annealing kinetics (Tautz, 1993; Tautz & Schlotterer, 1994).

In prokaryotes, genome size correlates with organismal complexity (Lynch & Conery, 2003). The increase in organismal complexity is associated with an increase in the number of genes. However, this is not true for eukaryotes, where the coding regions are less than what would be expected based on their organismal complexity (Bennett & Leitch, 2005). For example, coding regions in human DNA represent < 2% of the genome. In fact, the non‐genic regions of the genome increase with larger genome size, with the gene numbers being similar, except in the case of species with whole genome duplications such as maize, wheat, canola, or polyploids (Bennett & Leitch, 2005). Given that the selection pressure on intergenic regions of the genome is somewhat less than on genic regions, there is also more variability. This includes changes in the unit length of DNA repeats, which are among the most variable sequences in genomes.

DNA repeats are present throughout the genome, but they are especially abundant in certain regions such as centromeric and telomeric regions of the eukaryotic chromosome (Melters *et al*., 2013). Centromeric regions contain massive tandemly repeated arrays of sequences (Comai *et al*., 2017; Naish *et al*., 2021; Wlodzimierz *et al*., 2023). With recent telomere‐to‐telomere (T2T) assembly of sequences, we are also able to understand telomeric repeats, which reveal the complexity of telomeres (Miga *et al*., 2020; Belser *et al*., 2021; Logsdon *et al*., 2021; Deng *et al*., 2022; Nurk *et al*., 2022; Chen *et al*., 2023). For example, the average length of the Mo17 strains of maize telomere was *c*. 26 Kb with *c*. 3700 telomeric repeat (CCCTAA/TTACCG) sequences (Chen *et al*., 2023).

DNA repeats of smaller unit sizes (1–6 nucleotides) can also be seen in genic regions. Within genes, they can be present in both coding and noncoding regions. Coding regions are under functional constraints to ensure appropriate protein function and experience stronger selection pressure, compared to noncoding regions. Consequently, the distribution of DNA repeats differs between coding and noncoding regions of genes (Hancock, 1995; Toth *et al*., 2000). Often, repetitive DNA can flank unique non‐repetitive sequences. Such patterns are observed in ‘transposable elements’ (TE) (Feschotte, 2023). While TEs are distinct from typical DNA repeats, TEs can give rise to new repetitive elements and, in many ways, have similar patterns of regulation. It has been believed that TEs could be one of the key sources of microsatellite repeats in the genome (Kidwell & Lisch, 2000; Mestrovic *et al*., 2015; McGurk & Barbash, 2018; Zattera & Bruschi, 2022).

Another set of repetitive DNA includes duplicated genes (Magadum *et al*., 2013). Duplicated genes can occur in tandem or can also be dispersed across the genome after duplication. Either way, they result in copy number variation in genomes (Dolatabadian *et al*., 2017; Lye & Purugganan, 2019; Pos *et al*., 2021). Furthermore, there are specific protein motifs that are often repeated, and these include large repetitive sequences. For example, in the RNA polymerase V (NRPE1) protein, there is a 17 amino acid tandem repeat region that plays a critical role in its interactions with the ARGONAUTE protein, which appears to be conserved in NRPE orthologues (Trujillo *et al*., 2016). Similarly, there are other repeats such as leucine‐rich repeats, pentatricopeptide repeats, WD40 repeats, and many others, which play critical roles in protein function (Schaper & Anisimova, 2015). These protein tandem repeats are also reflected at the DNA level, to a certain extent due to multiple codons encoding the same amino acid.

Thus, DNA repeats can refer to multiple types of repetitive DNA. In this review, we mostly discuss the repetitive sequences that are primarily of microsatellite DNA in plants. We focus particularly on the molecular mechanisms that have been unearthed, which shed light on how repeat variability and repeat expansions contribute to phenotypic diversification and evolution. How variability in microsatellites has been exploited for mapping or as molecular markers for agricultural applications has already been extensively reviewed (Varshney *et al*., 2005; Garrido‐Cardenas *et al*., 2018; Taheri *et al*., 2018). Here, we draw upon comparisons and commonalities in some aspects of the other types of repeat sequences. For specific discussions on TEs, centromeric repeats, telomeric repeats, and copy number variation, readers are referred to these nice reviews (Lu *et al*., 2013; Dolatabadian *et al*., 2017; Janssen *et al*., 2018; Aksenova & Mirkin, 2019; Fresard *et al*., 2019; Lye & Purugganan, 2019; Talbert & Henikoff, 2020; Pos *et al*., 2021).

## II. Common allelic variation in repeat lengths vs rare repeat expansions

**Fig. 1:**
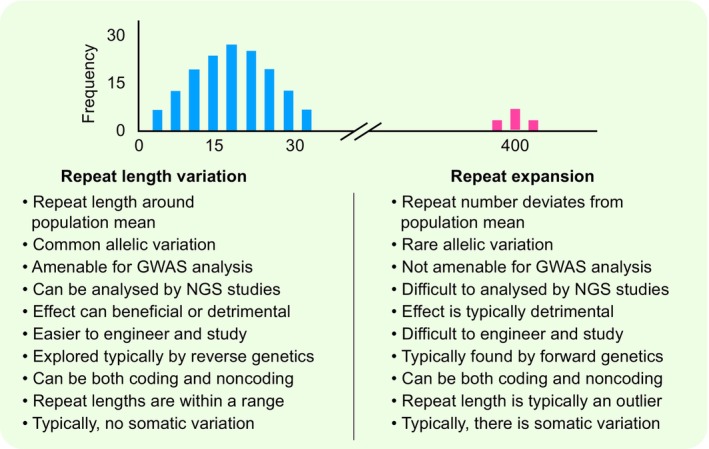
Differences between repeat length variations and repeat expansions. *x*‐axis refers to the number of copies of the repeat, and *y*‐axis refers to the frequency.

The repetitive satellite DNA can be sub‐categorised based on its unit length (Tautz, 1993). Repeats of 1–6 nucleotides are typically referred to as microsatellites or simple sequence repeats, which are very familiar to researchers as DNA markers typically used in gene mapping or population genetics studies. Microsatellite sequences are present in all organisms, from Archaea to humans, and they are among the most variable types of DNA sequences in the genome (Weber, 1990; Ellegren, 2004). In addition to the variability between individuals in a population, often, somatic variation within an individual can also be observed at microsatellites. Variability in the number of repeat units of microsatellites typically displays a continuous range in the population (Fig. 1). However, in rare instances, the number of repeat units exceeds this range observed in the population, as statistical outliers, and we define these as repeat expansions. Repeat expansion is a rare and extreme form of repeat length variation. However, repeat length variability and repeat expansion differ in several ways, with the repeat expansions typically identified through forward genetics based on their phenotypes and repeat length variability being explored through sequencing‐based approaches (Fig. 1).

Repeat expansions are well known in human biology, since they are associated with human disease (La Spada & Taylor, 2010; Malik *et al*., 2021). Expanded repeats can occur both in the coding and noncoding regions of the gene. If they are in the coding region, they encode repetitive stretches of amino acids, which can disrupt protein function. If the expansions occur in the noncoding regions of the gene, they tend to interfere with other processes, including gene silencing. Either way, repeat expansions tend to affect gene function and thus have been shown to be causal for several genetic diseases in humans. In this review, when we discuss repeat length variability, we refer to the variation that is seen in repeats that are within the range of most individuals of a population. We discuss repeat expansion separately, since it is a rare phenomenon. Expanded alleles can also vary, which refers to a distribution of repeat lengths around the mean length of the expanded allele within a population (Fig. 1).

## III. Repeats in plant genomes

As early as 1968, *C*_0_t curve analyses suggested that repetitive sequences are a common feature of genomes, including in plants (Britten & Kohne, 1968; Ingle *et al*., 1973). Subsequently, Flavell *et al*. (1974) showed that the variation in DNA content correlated with the proportion of variation in repeat content of diverse plant genomes, with larger genomes harbouring a higher proportion of repetitive DNA. It was also recognised in the 1970s that heterochromatic regions of rye plants harboured highly repetitive DNA. The first molecular characterisation of the repeat sequences came from rye plants, which turned out to be mostly interspersed repeats/minisatellite sequences (Bedbrook *et al*., 1980). Microsatellites, on the other hand, were originally described as ‘polypyrimidine tracts’ (Birnboim & Straus, 1975). Subsequently, Tautz and Renz showed via Southern hybridisation with probes that microsatellite repeats occur in diverse eukaryotes (Tautz & Renz, 1984). Soon after, plant geneticists picked up the usage of microsatellites as markers for mapping and marker‐assisted selection in plant breeding programmes (Condit & Hubbell, 1991; Morgante & Olivieri, 1993).

## IV. Variation in repeats contributes to phenotypic diversification

For a considerable period, repeat sequences (and a few other noncoding regions of the genome) were termed ‘junk’ DNA, which did not have any functional consequences. However, they were useful as molecular markers for genetic mapping or for characterising individuals in a population (Ohno, 1972; Doolittle & Sapienza, 1980; Orgel & Crick, 1980; Dimitri & Junakovic, 1999). Next generation sequencing approaches and associated reduction in sequencing costs have vastly increased the number of whole genome sequences. This availability along with the advances in computational analysis now allow us to decipher patterns of repeats in genomes. Analysis of intragenic repeats of *S. cerevisiae* suggested that they contribute to functional variability (Verstrepen *et al*., 2005). Similar observations were also seen in *Aspergillus* and *Neurospora* (Levdansky *et al*., 2007; Michael *et al*., 2007). These studies mainly focused on intragenic repeats that are in the coding region of the genes.

Typically, repeats in coding regions tend to be of a unit (e.g. triplet or hexamer), which allows them to withstand selection pressure without compromising the reading frame. Nevertheless, the repeats in the coding regions encode amino acids that are not reflective of a random distribution, suggestive of additional selective pressures. For example, analysis of *c*. 22 different proteomes revealed that only polyL, polyG, polyS, polyE, polyP, polyA, and polyQ could be observed at a relatively higher abundance, and most of the other amino acid repetitions in the proteome were not tolerated (Kumar *et al*., 2016). Similarly, cell culture studies in which imperfect repeat encoded polyQ and polyL constructs revealed that polyL is more toxic to cells than polyQ (Dorsman *et al*., 2002). It is also known that in humans, several genetic diseases are due to polyQ or polyA stretches in proteins (Winter *et al*., 2013; Darling & Uversky, 2017; Bunting *et al*., 2022; Tenchov *et al*., 2024). Perhaps due to general interest, functional studies on polyQ (or sometimes polyA) stretches are more common. While there are relatively fewer studies on poly amino acid stretches at a functional level, there are a few examples that highlight the importance of studies on plants on repeat variation. We will discuss some of them below.

## V. Variation in polyQ stretches modulate the function of ELF3 protein

Flowering time is a quintessential adaptive trait in plants that is regulated by both internal and external cues. Unsurprisingly, some of the earliest examples of phenotypic changes associated with variability in repeats in the coding region were found in proteins that play a role in flowering time regulation. One of the well‐researched examples of tandem repeat length variability in coding regions of genes with an effect on functional variation occurs in *EARLY FLOWERING 3 (ELF3)* in *Arabidopsis thaliana. ELF3* was originally isolated in a flowering time screen for mutants that show altered flowering behaviour (Zagotta *et al*., 1992). *elf3* mutants showed pleiotropic effects including impacts on flowering time, circadian clock, and hypocotyl elongation (Zagotta *et al*., 1992). Cloning of *ELF3* revealed that it encodes a novel protein that harboured three short runs of glutamine residues (Hicks *et al*., 2001). It was already recognised that there is variability in these stretches, with the Ws strain of *Arabidopsis* harbouring 16 residues of glutamine, compared to the reference strain Col‐0 that harboured 7 glutamine residues (Hicks *et al*., 2001). These glutamine stretches were caused by a CAA repeat with additional contribution from CAG codons. The repetitive nature at the amino acid level with differences in the triplets would indicate a potential selection at the amino acid level to keep the glutamine residues in those positions of the protein, as opposed to a simple repeat length variability caused purely by slippage in DNA synthesis or unequal cross over.

The functional relevance of this variation in polyQ stretches was explored by Christine Queitsch's group, where they showed that changing the polyQ lengths in the ELF3 protein had significant phenotypic consequences in a background‐dependent manner (Undurraga *et al*., 2012). In several studies, *ELF3* was identified as a potential Quantitative Trait Locus (QTL) associated with natural variation for the circadian clock (Tajima *et al*., 2007), shade avoidance (Coluccio *et al*., 2011), stochastic noise in the circadian clock (Jimenez‐Gomez *et al*., 2011), plant response to physical rhizosphere (Joseph *et al*., 2015) and thermo‐responsive growth (Box *et al*., 2015; Raschke *et al*., 2015). While some of this phenotypic variation is driven by other polymorphisms, the thermo‐responsive growth phenotype and a few other phenotypes have been clearly linked with the variability in the polyQ stretches in the protein.

## VI. Variation at polyQ influences biochemical properties of the ELF3 protein

**Fig. 2:**
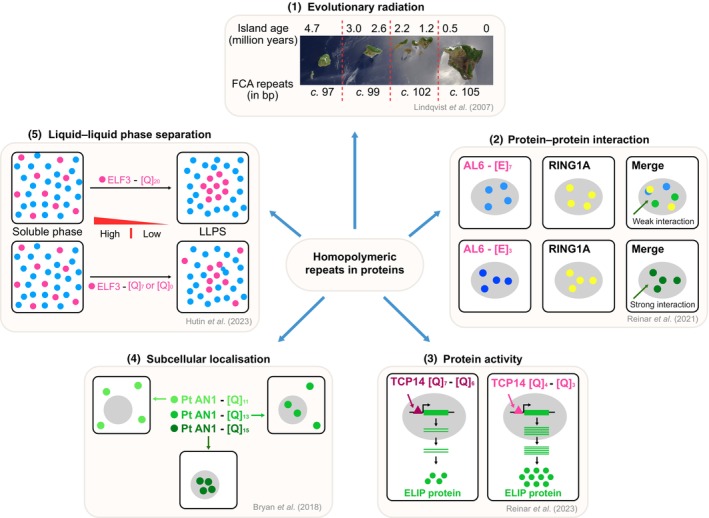
Variability in polyamino acid stretches in proteins has diverse functional impacts. (1) Evolutionary radiation – allelic variation in the polyQ stretch of the Hawaiian endemic mints of the Lamiaceae family shows a correlation between repeat length and geological gradients. (2) Protein–protein interactions in *Arabidopsis thaliana* – AL6 interacts with RING1A, where a polyE stretch of length 7 shows stronger interaction when compared to a polyE stretch of length 3. (3) Protein activity – TCP14 of *Arabidopsis thaliana*, a transcription factor with polyQ variation, regulates ELIP expression in a PolyQ‐length‐dependent manner. (4) Subcellular localisation – the polyQ stretches of the PtAN1 protein from *Populus trichocarpa* determine its localisation, with nuclear localisation at 11Q and cytosolic localisation at 15Q. (5) Liquid–liquid phase separation (LLPS) – the PrLD in ELF3 of *Arabidopsis thaliana* undergoes LLPS, with Q20 facilitating phase separation at lower temperatures compared to Q7 or Q0.

Although ELF3 is identified as a key protein mediating natural variation in diverse phenotypes, distinguishing the effect of the variation in polyQ and other polymorphisms has been difficult. Transgenic studies with proteins that differ only in the length of their polyQ stretches displayed clear phenotypic differences suggestive of functional impacts of the repeats (Undurraga *et al*., 2012). ELF3 contains a Prion‐like domain (PrLD), which is a type of intrinsically disordered region (IDR) present in proteins that lack a specific structure. This domain in the *Arabidopsis* ELF3 includes a polyQ region (Jung *et al*., 2020). The PrLD in ELF3 helps with liquid–liquid phase separation (LLPS) that leads to the formation of nuclear speckles, driving gene expression changes along with ELF4 (Fig. 2) (Herrero *et al*., 2012; Jung *et al*., 2020). Interestingly, the polyQ repeat lengths influenced the temperatures at which this LLPS occurs, with longer repeats (Q20) resulting in phase separation at lower temperatures when compared with shorter (Q7 or Q0) repeats (Hutin *et al*., 2023). Thus, the variability in glutamine stretches affected the biochemical properties of the ELF3 protein, and in the case of *Arabidopsis*, conferred sensitivity to temperature. However, it appears to be somewhat non‐essential in the context of thermal adaptation since variability in repeat length was not found to be correlated with environmental or geographic parameters (Zhu *et al*., 2024).

## VII. PFT1 – an example of evolutionary constraints on polyQ repeat number

A clear case of evolutionary selection on polyQ repeat length was described for PHYTOCHROME FLOWERING TIME 1 (PFT1, also referred to as MED25), a component of the mediator complex (Rival *et al*., 2014). The polyQ repeat in PFT1 is also an interrupted (CAA/CAG) rather than a perfect repeat, though the glutamine‐rich region in PFT1 is much larger than that of ELF3 (Rival *et al*., 2014). Constructs with both reduced and increased lengths of the repeats failed to complement, unlike the wild‐type allele (Rival *et al*., 2014). Analysis in *Arabidopsis* accessions revealed that the natural variation in repeat length at PFT1 is much smaller. Taken together, these data suggest that there are evolutionary constraints on the number of polyQ, to be precise in the context of PFT1, suggestive of selective pressures.

## VIII. Variation in homopolymeric stretches of amino acids can affect protein–protein interactions

Another great example of repeat length variability contributing to phenotypic variation and evolution came from a genome‐wide analysis of repeats in the coding regions of proteins in *Arabidopsis* (Reinar *et al*., 2023). Here, the authors tested the idea that homopolymeric stretches of amino acids typically result in IDRs, which lack structure and thus may influence protein–protein interactions. In the *Arabidopsis* proteome, the four most common homo‐polymeric stretches included polyQ, poly‐E, poly‐S, and poly‐D. Alfin‐like genes are Plant Homeodomain (PHD) ‐containing histone readers that bind to H3K4me2/3 marks and play a role in gene silencing. AL6 contains a polyE stretch that varies from 3 to 7 residues. Reinar *et al*. (2021) assayed constructs that differ in their polyE number in transient assays, which influenced both gene expression as well as protein–protein interaction (Fig. 2) (AL6–RING1A interaction).

Protein–protein interactions have also been suggested to be influenced by homopolymeric alanine (CCG repeat) stretches in plants (Li *et al*., 2023). In rice, expansion of agriculture in colder regions is associated with *COLD11*, which encodes RAD51A1, a protein involved in DNA repair. COLD11 has polymorphic alanine (PolyA) repeats that vary among the varieties, and the authors could demonstrate that lines that harbor a stretch of > 3 alanines display remarkable survival compared to lines that have < 3. Authors suggest that the alanine repeats may modulate protein–protein interactions (Li *et al*., 2023).

The *Arabidopsis* TCP family (teosinte branched 1, cycloidea & proliferating cell factor 1 family) of proteins contains IDRs and plays a role in the environmental response of plants. For example, TCP14 transcriptionally activates early light‐induced protein (ELIP) in high light stress (Sun *et al*., 2019). Using ELIP as a marker, authors tested whether variation in PolyQ in TCP14 can affect the efficiency of induction of ELIP and demonstrated that to be the case in transgenic experiments (Reinar *et al*., 2023).

How common are these impacts of the IDR regions? Reinar *et al*. (2023) developed and applied a computational pipeline to assess whether repeat‐containing proteins with predicted IDRs show that variability in homopolymeric stretches can potentially affect protein–protein interactions, which in turn, can influence protein function. Interestingly, these patterns displayed a correlation with climatic variables, thereby arguing for a role in adaptive evolution.

## IX. Variation in polyQ can affect the subcellular localisation of the protein

Though most of these studies are carried out in *Arabidopsis*, the impact of repeat variation in coding regions has also been shown in other plants. Variability in polyQ stretches has been shown to influence sub‐cellular localisation. In *Populus*, natural accessions display variation in polyQ stretches in the orthologue of the *Arabidopsis* ANGUSTIFOLIA (PtAN1) protein. Transient assays using protoplasts of *Populus* showed that PtAN1‐11Q was localised to the nucleus, and PtAN1‐15Q was mostly cytosolic (Fig. 2) (Bryan *et al*., 2018). This was reflected in both protein–protein interactions as well as in the expression levels of the downstream targets of PtAN1 (Bryan *et al*., 2018), potentially contributing to evolutionary divergence.

## X. Allelic variation in polyQ contributed to evolutionary radiation

The contribution of repeat length variation to evolution is nicely suggested in the radiation seen in Hawaiian mints (Lindqvist *et al*., 2007). There is variability in the polyQ stretch in the FCA protein, which was originally characterised in *Arabidopsis* as a gene regulating flowering time (Macknight *et al*., 1997). FCA protein has a polyQ stretch that varies from 0 to 17 glutamine residues in varied species. Lindqvist *et al*. (2007) unearthed the allelic variation in Hawaiian mint species and showed that repeat lengths correlate with the geological gradient (Fig. 2). In addition, they also displayed a correlation with reproductive traits suggestive of evolutionary selection. Here again, statistical network analysis suggested variation in protein–protein interactions.

In summary, genome‐wide analysis presents statistical patterns with repeat length variation resulting in homopolymeric stretches of amino acids that appear to impact protein–protein interaction (PPI) networks, and this perturbation of the PPI networks appears to affect their functionality, resulting in functional consequences, presenting a fertile ground for evolutionary selection to operate (Fig. 2).

## XI. Repeats in noncoding regions

Many tandem repeats occur in the noncoding regions of genes and the intergenic regions, including promoters and enhancers. Let us first examine the repeat length variability in the genic repeats that occur in introns and UTRs, which are the noncoding regions of genes. The evolutionary constraints on the noncoding regions are less compared to the coding regions. Accordingly, the proportion of variable tandem repeats in the noncoding regions of the gene is much higher when compared to those in the coding regions (Press *et al*., 2018). Studies in the human system reveal that tandem repeat length variability is associated with differences in gene expression (Gymrek *et al*., 2016; Fotsing *et al*., 2019; Wright & Todd, 2023). In plants, too, we can observe an effect of tandem repeat length variability on gene expression (Press *et al*., 2018; Reinar *et al*., 2021).

Repeats in both coding and noncoding regions have a functional impact. While the repeats in coding regions impacted protein–protein interactions, protein activity, subcellular localisation, and LLPS (Fig. 2), repeats in noncoding regions affect gene expression through other mechanisms (Reinar *et al*., 2021). Reinar *et al*. (2021) modelled gene expression as a function of tandem repeat length and found several repeats to have a significant impact on gene expression. Reinar *et al*. (2021) reasoned that if tandem repeats indeed influence gene expression, they will show a distance bias, with the repeats near the genes likely having a higher effect. Indeed, they identified a general effect of tandem repeats on gene expression in *Arabidopsis*.

**Fig. 3:**
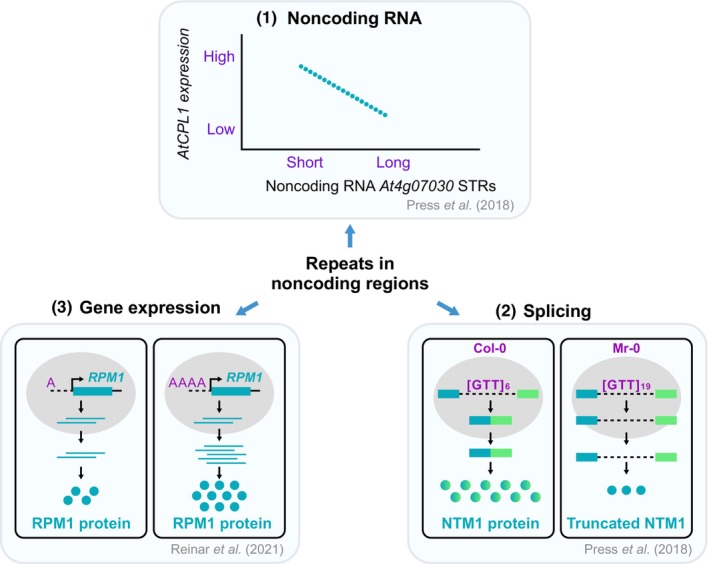
DNA repeats in noncoding genic regions can affect distinct cellular processes. Examples shown are from *Arabidopsis thaliana*. (1) Noncoding RNA – the STR variation in the noncoding RNA *At4g07030* is correlated with the expression of *AtCPL1*. (2) Splicing – intronic repeats of 6 or 19 GTTs at the *NTM1* locus has an impact on the intron retention, leading to a truncated protein in the Mr‐0 accession of *Arabidopsis thaliana*. (3) Gene expression – mononucleotide stretch variability at the *RPM1* promoter has an impact on the levels of RPM1 protein produced in *Arabidopsis thaliana*.

An alternative but classical approach was taken by Press *et al*. (2018) where they analysed the effect of tandem repeats on gene expression by eQTL analysis. Even with a small sample size, they were able to identify eQTLs, once again arguing for the ability of repeats to potentially modulate gene expression. Interestingly, one of the associations was with a repeat in the noncoding RNA (At4g07030), which was associated with the expression of *AtCPL1* (Fig. 3) (Press *et al*., 2018). Here again, *AtCPL1* is near the repeat, suggesting that the distance makes a difference to the effects of tandem repeats on gene expression.

A second mechanism through which noncoding repeats make a difference to gene regulation occurs at the level of splicing, particularly if the repeats are present in the introns. For example, at the *NTM1* locus, there are *c*. 6 GTT repeats in an intron, and in natural accessions of *Arabidopsis*, the longest allele has *c*. 19 repeats. Reverse transcription polymerase chain reaction analysis suggested this allelic variation negatively affects splicing, resulting in intron retention (Press *et al*., 2018). This repeat is physically close to a splice acceptor site. Therefore, it is feasible that the expansion perhaps makes the branch point sequence too far away from the acceptor site, which in turn might lead to defective splicing (Fig. 3). The impact of tandem repeats on splicing was further elaborated by Zhang *et al*. (2025) who examined the impacts of tandem repeats on the usage of individual splice sites (Dent *et al*., 2021). Analysis of the proximity of the tandem repeats that are associated with differential splice‐site usage revealed that these repeats were enriched in the noncoding regions (introns and UTRs) of the genes, a pattern that was also seen with SNPs that are associated with differential splice site usage in genome wide association studies in *Arabidopsis* (Dent *et al*., 2024; Zhang *et al*., 2025).

With the rise of pangenomes, the association between non‐coding repeat numbers and gene expression has become clearer. For example, an association analysis with tandem repeats in rice revealed that the *TRGW6* gene harbors one GT dinucleotide repeat in Nipponbare, while other strains harbored multiple GT repeat units at the *TRGW6* promoter (He *et al*., 2024). Authors showed an association between grain width and GT repeat number. Subsequent CRISPR‐cas9 edited crops revealed that a single additional unit of GT increased grain width, presumably by modulating the expression levels of the *TRGW6* gene (He *et al*., 2024).

In summary, repeats in the noncoding genic regions appear to have an impact on noncoding RNA, gene expression, and splicing (Fig. 3). The exact mechanisms of these regulations are currently unclear. However, it is presumed that they may involve different processes. First, variation in noncoding repeats could affect gene expression, perhaps by affecting the efficiency of binding of transcriptional regulators. Second, they could influence the spliceosomal machinery, potentially affecting the binding kinetics of the spliceosomal components. Third, repeats in the noncoding RNA could affect its function, perhaps by RNA modifications, which could affect downstream processes (Fig. 3).

## XII. Repeat expansions

As we defined earlier, for consistency and to avoid confusion, we define that extreme variation in repeat lengths, which are not common allelic variations, as repeat expansions. While repeat length variability was discovered through sequencing approaches, most of the repeat expansions were found through forward genetic approaches, mostly in human disease. Several neurogenetic disorders, such as Huntington disease, Friedreich's ataxia and Fragile X syndrome, are caused by repeat expansions in genes (Verkerk *et al*., 1991; Huntington Consortium, 1993; Campuzano *et al*., 1996). Originally, since these expansions were seen primarily in humans, researchers at some stage even considered repeat expansions as a human‐specific phenomenon (Sutherland & Richards, 1995). However, it turned out to be simply due to the higher number of studies that were performed at the population level, as forward genetic studies on the population level in *Arabidopsis* too resulted in the discovery of a repeat expansion (Sureshkumar *et al*., 2009).

Repeat expansions present a different type of challenge to study as opposed to studying repeat length variability and its impacts (Fig. 1). As we have seen earlier, repeat length variability within the normal range is easily amenable to experimentation, and thus, most of the analysis is reverse genetics, and transgenic approaches are really feasible. By contrast, engineering large repeat expansions that are typically seen in the noncoding regions proved to be difficult (Al‐Mahdawi *et al*., 2004, 2006; Anjomani Virmouni *et al*., 2015; Kalef‐Ezra *et al*., 2023). Given that most of the natural repeat expansions have been discovered in humans, there have been attempts to generate the expansions with transgenic approaches in other systems. This worked reasonably well for the expansions in coding regions, for example, in flies (Bilen & Bonini, 2005). However, PCR products that harbor large, expanded repeats are hard to clone and generally lead to loss of repeats in *Escherichia coli*. Even estimating the exact size of the repeat expansions in noncoding regions remained difficult. For example, initial studies that discovered the repeat expansion as late as in the case of *Arabidopsis* in 2009 employed Southern blot analysis to get an idea of the repeat length in expanded alleles (Sureshkumar *et al*., 2009). Recent advances in long‐read sequencing present an opportunity to identify expansions more easily as opposed to their earlier discoveries through forward genetic approaches. In fact, in the last 5 years, several repeat expansion diseases were found due to these advances (Ishiura *et al*., 2023; Leitao *et al*., 2024). However, the utilisation of these approaches to plant genomes or a focus on uncovering expanded repeats has been minimal.

In humans, most expansions in coding regions tend to be from polyQ stretches. As we discussed above, even in plants, several polyQ stretches are associated with phenotypic variation. However, in most of these cases, one would consider them as variants within a normal range (i.e. repeat length variation) as opposed to expansions (i.e. variation beyond what is normal in the population, an outlier). In addition, several of these polyQ stretches are made up of imperfect repeats containing both CAA and CAG codons, which suggests evolutionary selection at the level of protein functionality rather than a by‐product of polymerase slippage.

## XIII. A case of repeat expansion in plants

**Fig. 4:**
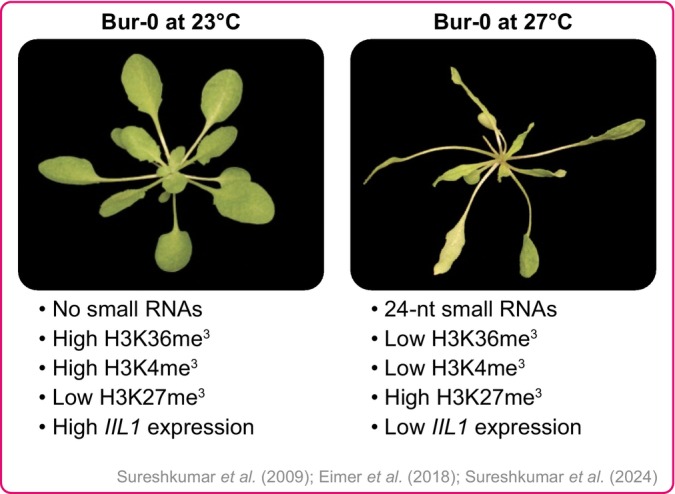
Intronic repeat expansion at *IIL1* leads to a temperature‐dependent growth defect in *Arabidopsis thaliana*. Bur‐0 accessions harbour a GAA/TTC repeat expansion at the *IIL1* locus. This repeat expansion at 27°C leads to 24‐nt small RNAs, which via the RdDM pathway, with deSUMOylase FUG1, histone reader AL3, and LHP1 switch the status of the *IIL1* locus from the active state with H3K36me^3^ marks to the repressed state with H3K27me^3^ marks. This leads to a reduction in *IIL1* expression, leading to a growth defect.

The discovery of a repeat expansion in plants was a serendipitous discovery from a forward genetic screen. In a screen for natural variation in temperature sensitivity, researchers observed that the Bur‐0 accession of *Arabidopsis* from Ireland failed to grow at higher temperatures (27°C) but grew normally at lower temperatures (23°C) in short‐day conditions (Fig. 4). The differential growth at varied temperatures allowed mapping of this growth defect. Genetic mapping revealed that the *‘irregularly impaired leaves (iil)’* phenotype seen at higher temperatures was due to a GAA/TTC repeat expansion in the intron of the *ISOPROPYL MALATE ISOMERASE LARGE SUBUNIT 1 (IIL1)* gene, which encodes an enzyme required for leucine/glucosinolate biosynthesis in *A. thaliana* (Sureshkumar *et al*., 2009).

One of the advantages of the plant system, particularly in the context of repeat expansions, is the ability to do genetic analysis, which is often difficult or tricky to do in animal systems. For example, unlike the human disease examples, the Bur‐0 repeat expansion could be conclusively shown to be the underlying cause for the observed phenotype through precise genetic mapping to a very short interval (16.7 Kb) and spontaneous revertant screens (Sureshkumar *et al*., 2009). Expanded repeats often display genomic instabilities. This was exploited in the context of Bur‐0, where 30 000 plants were grown at higher temperatures, and natural suppressors that displayed normal growth were isolated. When repeat expansion was assessed in these plants, interestingly, all normal‐looking plants had reduced repeat lengths, which conclusively showed that the repeat expansion was responsible for the observed phenotypes (Sureshkumar *et al*., 2009).

The repeat expansion at *IIL1* was intronic, and like the intronic repeat expansion observed in Friedreich's ataxia (FRDA), where a GAA/TTC repeat expansion at the 3^rd^ intron of the *FRATAXIN (FXN)* gene leads to a reduction in its expression, leading to reduced FXN protein levels. In *Arabidopsis*, transgenic increase of *IIL1* expression abolished the *iil* phenotype, demonstrating that the reduction in gene expression is responsible for the observed phenotype (Sureshkumar *et al*., 2009).

In FRDA, reduction in gene expression is attributed to epigenetic silencing of the locus (Herman *et al*., 2006; Al‐Mahdawi *et al*., 2008; Rai *et al*., 2010; Kim *et al*., 2011; Sandi *et al*., 2013; Yandim *et al*., 2013). Therefore, it naturally raised the question of whether the *IIL1* repeat expansion is also affected via epigenetic silencing. ChIP experiments revealed that typically there is an enrichment of H3K36me^3^ histone marks at the *IIL1* locus in the reference strain (i.e. lacking repeat expansion), and in the genotypes that lack the expanded allele (Eimer *et al*., 2018). However, in the presence of the repeat expansion, and at the temperature (27°C) where one observes the *iil* phenotype, there was an epigenetic switch with the enrichment of H3K27me^3^ marks associated with a reduction in *IIL1* expression (Fig. 4) (Eimer *et al*., 2018). Although epigenetic silencing at FRDA has been known for a long time, the pathway through which expanded repeats lead to epigenetic silencing remains largely unknown in FRDA (Chutake *et al*., 2014, 2016; Nageshwaran & Festenstein, 2015). The remarkable genetic power of *Arabidopsis* allows for the dissection of these pathways at a molecular level.

## XIV. Repeat expansions can give rise to small RNAs, which lead to epigenetic silencing

One of the questions that arises is whether repeat expansions can act as templates for small RNA production. Indeed, some reports suggested that triplet repeats can form hairpins and thus have the potential to be targeted by DICERs (Krol *et al*., 2007). This indeed turned out to be the case in the *Arabidopsis* repeat expansion, where there was an increase in the 24 nt siRNAs that map to the *IIL1* locus, specifically at 27°C, where the *iil* phenotype was clearly observed (Eimer *et al*., 2018). Once again, plants provide an excellent opportunity here, since the processing of small RNAs of different lengths is carried out by different Dicer‐like (DCL) enzymes, which allow experimentally testing the necessity of the small RNAs in epigenetic silencing. Indeed, knocking down *DCL3*, which is required to produce 24 nt small RNAs, suppressed the *iil* phenotype. Subsequent transgenic knockdown experiments as well as second‐site suppressor screens showed that the RNA‐dependent DNA methylation (RdDM) pathway is critical for epigenetic silencing caused by expanded repeats (Eimer *et al*., 2018; Sureshkumar *et al*., 2024).

Studies in plants also provided some novel insights that are currently being explored in a translational manner in our group to understand FRDA. A genetic suppressor screen of the *iil* phenotype revealed a role for deSUMOylation, a post‐translational modification. Mutations in *FOURTH UBIQUITIN‐LIKE GENE CLASS 1 (FUG1)* suppressed the epigenetic silencing of the *IIL1* locus, suggestive of its requirement for gene silencing (Sureshkumar *et al*., 2024). Interestingly, the closest homolog of FUG1 in human systems is the Sentrin‐like proteases (SENPs), of which some are known to deSUMOylate HETEROCHROMATIN PROTEIN 1 (HP1), which is a required protein for epigenetic silencing in FRDA (Maison *et al*., 2012; Romeo *et al*., 2015). Studies are currently underway to test whether SENPs play a role in epigenetic silencing in FRDA.

The repeat expansion not only presents an opportunity to study DNA repeats and their mechanisms (Fig. 4), but it also allows us to infer generalisable principles in biology and reveal cryptic functions and phenotypes. For example, among the 5 different deSUMOylases in *Arabidopsis*, and while the functions of other deSUMOylases are known, FUG1 remained an uncharacterised protein, until its function was revealed recently (Morrell & Sadanandom, 2019; Clark *et al*., 2022). While mutations in *fug1* do not lead to a clear phenotype in the reference strain Col‐0, there is a striking suppression of the *iil* phenotype in the Bur‐0 background (Sureshkumar *et al*., 2024). Similarly, two hybrid interaction studies revealed that the SUMO protease FUG1 interacts with a histone reader ALFIN LIKE‐3 (AL3) that binds to H3K4me^3^ marks. On the other side, AL3 interacts with LIKE HETEROCHROMATIN PROTEIN 1 (LHP1), which binds to H3K27me^3^ marks and spreads that across the chromatin to cause gene silencing (Fig. 4) (Sureshkumar *et al*., 2024). While this may be revealed through the analysis of the repeat expansion phenotype, this study illuminated a generalisable principle of how post‐translational modifiers may enable interactions between histone readers and the polycomb repressive complex to cause gene silencing (Sureshkumar *et al*., 2024). It is interesting to note that the same pathway is also required for epigenetic silencing of TEs in plants (Cao & Jacobsen, 2002; Pontes *et al*., 2006). Thus, at some level, repeat expansion in noncoding regions and TEs and other repeats that are silenced, appear to share mechanisms through which gene silencing occurs in plants.

## XV. Evolution of repeats and repeat expansions

The origin of the repeats themselves perhaps could be traced to TEs. Transposable elements play a key role in shaping evolution by modifying phenotypes through a variety of mechanisms. The ability of TEs to relocate across the genome acts as a source of genetic variation, nicely demonstrated, for example, in enabling rapid adaptation as shown for *Capsella rubella* (Niu *et al*., 2019). Similarly, variation in TEs also modulates quintessential adaptive traits such as flowering time and stress response (Hassan *et al*., 2023; Tossolini *et al*., 2025). The genomic loads of TEs vary among different species, populations, or individuals (Jiang *et al*., 2024). It has been demonstrated that demographic history, active TEs, and causal genes could account for the variation of TE load in Arabidopsis and its relatives (Agren *et al*., 2014). As such, it is conceivable that repetitive DNA that arises from TEs also plays a critical role in adaptive processes and thus is an important agent for evolution. The functional implications that we have reviewed here provide ample evidence for their role in evolutionary processes.

We suggest that after the origin of the repeats, perhaps from TEs or other processes due to polymerase slippage, unequal crossover, and even gene duplication mechanisms, repeat expansions can arise. In addition, repeat regions display an excessive rate of mutations (Zhang *et al*., 2025). The evolutionary selection pressures on repeats appear to vary based on whether they are in the coding or noncoding regions of the genes. In coding regions, maintaining the functionality of the protein becomes a key constraint in achieving variability. By contrast, in the noncoding region, selective pressures are independent of the functionality of the protein but are rather related to its levels, and perhaps may also be influenced by the repeat expansion being able to sequester some proteins. Although such examples have not yet been shown clearly in the context of plants.

DNA repeats and their variability in the normal range have the potential to be common allelic variations that can drive phenotypic variation on a continuous scale. On the other hand, repeat expansions are generally rare allelic variations. Therefore, normal techniques such as Genome Wide Association Studies (GWAS) or simple sequencing approaches would generally be ineffective in identifying expansions (Fig. 1). Clearly, long‐read sequencing has helped the discovery of several repeat expansion diseases, which is not yet the case in plants, and thus the expansions themselves are much harder to detect. However, it appears to be feasible, since a T2T assembly of the maize genome revealed that there are long stretches of trinucleotide repeat sequences that extend up to hundreds of Kb (Chen *et al*., 2023).

One of the key questions that remains unanswered is what makes a particular repeat undergo expansion. A simple scan of the genome would suggest that several genes have repeats and perhaps display some level of variation; the repeat expansions themselves are quite rare. One approach that has been attempted is to use Bayesian modelling to ask whether any genomic patterns exist among repeats that undergo expansions. While these approaches have not yet led to fruitful outcomes, with the development of computational tools and artificial intelligence‐based approaches, one may be able to decipher specific patterns among DNA repeats that undergo expansions. Given the intrinsic biochemical nature of repeat expansions, a combinatorial analysis of repeat expansions independent of the species may also be helpful in unravelling the patterns, which in turn would allow us to uncover repeats that could undergo expansions and thus may be associated with severe phenotypes.

## XVI. DNA repeat variability and repeat expansions – an underexplored resource of genetic variation

Genome‐wide association analysis generally may not include variation seen in repeat sequences, and thus, their contribution is generally missed out. While repeat expansions are still harder to capture, next‐generation sequencing studies on a large scale, such as the 1000 Genomes Project, UK Biobank project, Drosophila Genotype Reference Panel, and 1001 Genomes Project, etc., have developed massive resources that are amenable for exploring repeat variability in greater detail. The 1001 Genomes Project in *Arabidopsis*, for example, presents an excellent opportunity to explore the long‐read sequencing data for repeat expansions (Consortium *et al*., 2024). These then allow one to test the impacts of DNA repeat variability on phenotypes, as we discussed above. Almost no systematic analysis of repeats other than their use as markers has been carried out in any crops or other plant species, which we believe would be an excellent opportunity as more and more genome sequences become available. The worthiness of such approaches is evidenced by the fact that at least in humans, some of the missing heritability could be attributed to tandem repeat sequences (Gymrek & Goren, 2021; Mukamel *et al*., 2021). By analyzing exome sequences of UK Biobank participants, Mukamel *et al*. (2021) revealed substantial associations of repeat variability with diverse phenotypes. While some such studies are beginning to emerge in *Arabidopsis* and rice (Reinar *et al*., 2021; Li *et al*., 2023; He *et al*., 2024; Zhang *et al*., 2025), it appears that the time is ripe for such studies to be carried out in crop species, which may provide crucial clues on diverse plant phenotypes including their environmental responsiveness, with an ultimate impact on agricultural productivity.

The frequency of DNA repeat length variation in plants is somewhat comparable to that in humans, though there is variation between different species, with larger genomes perhaps having a bit of a higher proportion of diverse repeats. There is no *a priori* reason to believe that plant proteomes or plant DNA repeats are likely to display distinct and different consequences. If any, we would argue, plants, due to their sessile nature and their ability to withstand and manage diverse environments, are more likely to use any possible genetic variation to their advantage. Perhaps active TEs, compared to animals, may present additional opportunities for plants to originate, maintain, and evolve novel repetitive sequence‐mediated genetic variation. Therefore, we would propose that there is plenty of phenotypic variation that is yet to be discovered in plants that are associated with changes in simple sequence repeats. DNA repeat variability is likely to contribute to adaptive environmental responses, which may not be uncovered in traditional phenotypic analysis experiments.

While DNA repeat variability has been seen to contribute both as a negative and positive impact on functionality/fitness, DNA repeat expansions generally appear to have a negative impact on evolutionary fitness, particularly in humans with repeat expansion diseases. However, it is also feasible that only such negative effect expansions are amenable to discovery through forward genetic approaches. In the plant example of a repeat expansion, a survey at the Burren region in Ireland, where the original Bur‐0 strain that harbours the repeat came from, one can still find accessions with expanded repeats (Tabib *et al*., 2016). It is feasible that the negative effect of the repeat, which is seen primarily at high temperatures, may not cause an effect; it is also conceivable that the repeat expansion provides an unclear or unknown advantage. For example, an indirect consequence of repeat expansion is an impact on flowering time, which is clearly delayed, with the spontaneous revertant displaying an early flowering phenotype, which could have positive impacts (Tabib *et al*., 2016). Thus, plants provide a unique opportunity to address some of these questions, which may be impossible to address in the human system.

Current techniques for DNA sequencing and computational tools have increased our ability to uncover DNA repeat variability at the population scale very well. However, DNA repeat expansions, on the other hand, are still harder to get their hands on easily. While forward genetics has been the key in identifying DNA repeat expansions, analysis at a larger scale is only feasible in a reverse genetics approach. This is an ongoing development, which will increase our ability to identify the expansions. Optical genome mapping appears to provide some advantages and promise in detecting expansions, but the scalability of this approach is still unclear (Dremsek *et al*., 2021; Facchini *et al*., 2023). With the increasing ability to sequence long stretches of DNA through advanced sequencing technologies, we predict this problem may be solved. With CRISPR technology, we can perturb repeat expansions to alter phenotypes caused by repeat expansions. While this is being attempted in the context of gene therapy in humans, one would expect that it would be a matter of time before the same approach is used, perhaps more easily in plants to alter phenotypes. Future years hold great promise for functional analysis of simple sequence repeats and their impacts on plants, which will have significant impacts on agricultural productivity.

## Competing interests

None declared.

## Author contributions

SS: writing of the manuscript and synthesis of information. AC: preparation of figures and writing of the manuscript. YG: contributed to the synthesis of information. SB: conceptualised, coordinated and wrote the manuscript.

## Disclaimer

The New Phytologist Foundation remains neutral with regard to jurisdictional claims in maps and in any institutional affiliations.
